# Insights into battles between *Mycobacterium tuberculosis* and macrophages

**DOI:** 10.1007/s13238-014-0077-5

**Published:** 2014-06-18

**Authors:** Guanghua Xu, Jing Wang, George Fu Gao, Cui Hua Liu

**Affiliations:** 1 CAS key Laboratory of Pathogenic Microbiology and Immunology, Institute of Microbiology, Chinese Academy of Sciences, Beijing, 100101 China; 2 School of Life Sciences, Anhui University, Hefei, 230039 China

**Keywords:** *Mycobacterium tuberculosis*, macrophages, necroptosis, apoptosis, autophagy, tumor necrosis factor (TNF), type I Interferons (IFNs)

## Abstract

As the first line of immune defense for *Mycobacterium tuberculosis* (Mtb), macrophages also provide a major habitat for Mtb to reside in the host for years. The battles between Mtb and macrophages have been constant since ancient times. Triggered upon Mtb infection, multiple cellular pathways in macrophages are activated to initiate a tailored immune response toward the invading pathogen and regulate the cellular fates of the host as well. Toll-like receptors (TLRs) expressed on macrophages can recognize pathogen-associated-molecular patterns (PAMPs) on Mtb and mediate the production of immune-regulatory cytokines such as tumor necrosis factor (TNF) and type I Interferons (IFNs). In addition, Vitamin D receptor (VDR) and Vitamin D-1-hydroxylase are up-regulated in Mtb-infected macrophages, by which Vitamin D participates in innate immune responses. The signaling pathways that involve TNF, type I IFNs and Vitamin D are inter-connected, which play critical roles in the regulation of necroptosis, apoptosis, and autophagy of the infected macrophages. This review article summarizes current knowledge about the interactions between Mtb and macrophages, focusing on cellular fates of the Mtb-infected macrophages and the regulatory molecules and cellular pathways involved in those processes.

## INTRODUCTION

Approximately one-third of the population in the world are infected with *Mycobacterium tuberculosis* (Mtb), If untreated, one in ten latent infection will progress into active tuberculosis (TB) disease with about 50% mortality rate. According to the WHO report, an estimated 8.6 million people developed TB, and 1.3 million died from the disease (including 320,000 deaths among HIV-positive TB patients) in 2012. Furthermore, the global estimate of the burden of MDR-TB was 300,000 cases among notified TB patients in 2012. India and China were the two countries estimated to have the largest numbers of MDR-TB patients (both over 50,000) (Bogdan, 2000; Gonzalez-Navajas et al., 2012; WHO, 2013). As an intracellular pathogen, Mtb lives in macrophages, which act as the first line of immune defense for Mtb by clearing the pathogen. In the progress of cellular immunity against Mtb, macrophages also function as antigen presenting cells, in which the antigens of Mtb are degraded into immunogenic polypeptides and presented to T cell by major histocompatibility complex to trigger adaptive immunity. In turn, through long battles with host, Mtb has developed a plethora of strategies to counteract the bactericidal activities of the host immunity, thereby successfully establishing a niche for long-term survival within macrophages (Jayachandran et al., 2013). The battles between Mtb and macrophages have been constant since ancient times and become more complicated with the appearance of drug-resistant Mtb, especially the multidrug-resistant (MDR) and extensively-drug resistant (XDR) Mtb (Guirado et al., 2013; Warner and Mizrahi, 2013). In this review, we summarize current knowledge about the interactions between Mtb and macrophages, focusing on cellular fates of the infected macrophages and the regulatory molecules and cellular pathways involved in those processes.

## MACROPHAGE CELLULAR FATES DURING MTB INFECTION

Different cellular fates of Mtb-infected macrophages are of great importance as the death modality influences the outcome of infection. During Mtb infection, several forms of cellular fates have been observed such as necroptosis, apoptosis and autophagy, among which apoptosis and autophagy have been recognized as innate macrophage defense mechanisms (Behar et al., 2010; Behar et al., 2011; Bradfute et al., 2013; Du et al., 2013). Apoptotic death reduces the viability of different mycobacterial species, including Mtb. The finding that macrophages infected with virulent Mtb undergo necroptosis, whereas those infected with attenuated mutant strains of Mtb undergo apoptosis, suggests that wild type Mtb actively inhibits apoptosis. During Mtb infection, autophagy represents not only an antimicrobial mechanism for the clearance of the intracellular pathogens, but also prevents excessive inflammation, avoiding the adverse effects on host (Bradfute et al., 2013; Yu et al., 2013). Results from a genome-wide analysis of the host intracellular network that regulates survival of Mtb showed that those factors predominantly function through the regulation of autophagy (Kumar et al., 2010). In addition, several studies revealed genetic associations between TB and host genes involved in autophagy such as the *irgm* gene (Songane et al., 2012).

During Mtb infection, multiple molecules and their associated signaling pathways in macrophages are involved in the regulation of cellular fates of host cells. After being inhaled, Mtb can bind to phagocytic receptors and enters resident alveolar macrophages recruited from the bloodstream (Bhatt and Salgame, 2007). Macrophages also express Toll-like receptors (TLRs) that recognize conserved pathogen-associated-molecular patterns (PAMPs) on Mtb to mediate the production of cytokines such as tumor necrosis factor (TNF) and type I Interferons (IFNs) (Bhatt and Salgame, 2007; Hayashi et al., 2001; Killick et al., 2013). TNF can induce both apoptosis and necroptosis. Type I IFNs can control Mtb growth as well as cause damage to host cells by induction of either apoptosis or necroptosis. In addition, accumulating studies have shown that Vitamin D plays a vital role in host defense against Mtb by regulating innate immunity in human macrophages, mainly through induction of cathelicidin and autophagy (Liu et al., 2006; Shin et al., 2010).

## TNF CAN INDUCE APOPTOSIS AND NECROPTOSIS OF MTB-INFECTED MACROPHAGES

In Mtb infected macrophages, diverse components derived from mycobacterial cell walls could stimulate TNF production through TLR2-mediated signaling pathway (Underhill et al., 1999). TNF acts on TNF receptors, which exist in soluble or membrane-bound forms, and further induces host cell apoptosis (Flynn and Chan, 2001). TNF activates caspase 8-mediated extrinsic cell death pathway involving the kinases ASK1, p38 and c-Abl (Kundu et al., 2009), which lowers the spread of mycobacteria (Behar et al., 2011). Extracellular vesicles derived from apoptotic Mtb infected macrophages carry antigens and can be engulfed by uninfected dendritic cells, then those antigens are further presented to CD8^+^ T cells by MHC-I and CD1, causing activation of T lymphocytes and triggering adaptive immunity (Schaible et al., 2003). Virulent strains such as Mtb H37Rv induce less macrophage apoptosis than avirulent or attenuated Mtb strains such as Mtb H37Ra by activating the release of membrane-bound TNFR2 as the soluble form to evade TNF-dependent apoptosis (Balcewicz-Sablinska et al., 1998; Keane et al., 2000; Rakotosamimanana et al., 2013), and by increasing the expression of Mcl-1 protein that is a member of anti-apoptotic B-cell lymphoma/leukemia 2 (Bcl-2) family (Abdallah et al., 2011; Chen et al., 2001; Sly et al., 2003). The Bcl-2 family proteins, which are located in the outer membrane of mitochondria, can block the release of cytochrome c from mitochondria (Chen et al., 2001). In addition, several Mtb gene products can inhibit host cell apoptosis. For example, *nuoG* encodes one subunit of the type I NADH dehydrogenase in Mtb, and over-expression of Mtb *nuoG* in the apoptosis-inducing species *M. kansasii* leads to inhibition of apoptosis of infected human and murine macrophages (Velmurugan et al., 2007). In addition, Mtb can neutralize NOX2-derived reactive oxigen speices (ROS) in order to inhibit TNF-mediated host cell apoptosis via a NuoG-dependent mechanism (Miller et al., 2010). Similar function can also be observed for PknE and SecA2. Deletion of *pknE* results in a mutant that was more susceptible to NO exposure, and the Mtb *pknE* mutant induces higher level of apoptosis than wild type strain (Jayakumar et al., 2008). *secA2* deletion mutant of Mtb induces more apoptosis than wild type Mtb in infected macrophages (Hinchey et al., 2007).

**Figure 1:**
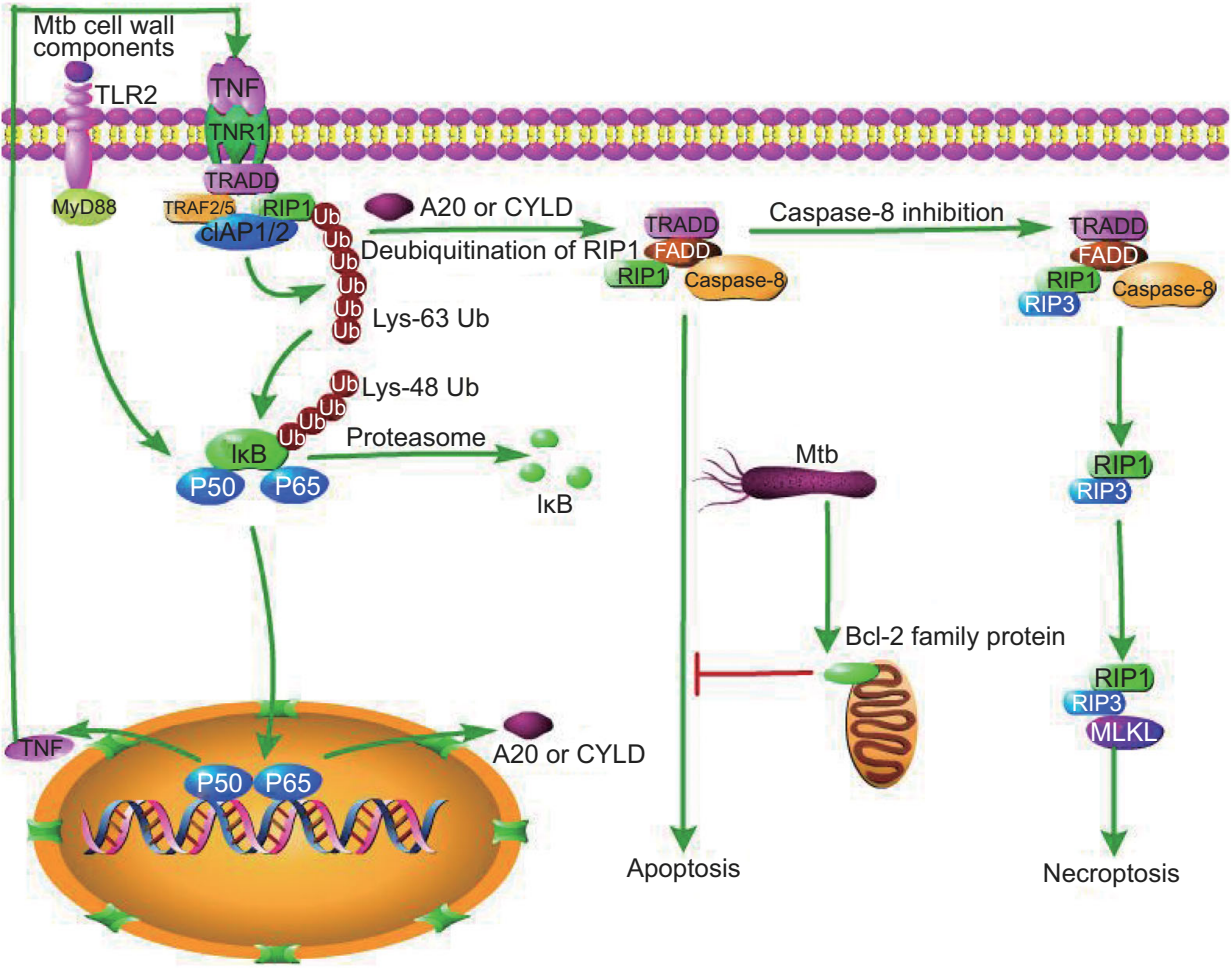
**TNF can induce the apoptosis and necroptosis of Mtb-infected macrophages**. Mtb cell wall components cause stimulation of TLR2, which leads to translocation of NF-κB to nucleus and induces expression of TNF through a myeloid differentiation factor 88 (MyD88)-dependent manner. As TNF binds to TNFR1, TRADD binds to RIP1 and TNFR1 and cIAP1/2 to form complex I. Lys-63-linked polyubiquitination of RIP1 or TRAF2 by cIAPs results in IκB Lys 63-linked proteasomal degradation. Translocation of NF-κB into nucleus induces transcription of its target genes including A20 and CYLD. A20 and CYLD deubiquitinate RIP1,which switches complex I to complex II, then apoptosis is initiated. Upon inactivation of casepase-8, complex III is formed and RIP1 recruits RIP3, and then RIP3 recruits MLKL, ultimately leading to necroptosis. Mtb can also inhibit apoptosis by up-regulation of Bcl-2 family protein

TNF is also known to be a potent inducer of mitochondria ROS. High TNF production induces mitochondrial ROS in infected macrophages through receptor interacting protein 1 (RIP1)–receptor interacting protein 1 (RIP3)–mixed lineage kinase domain-like protein (MLKL)–phosphoglycerate mutase family member 5 (PGAM5)–dynamin-related protein-1 (Drp1)-dependent pathways. While initially increasing macrophage microbicidal activity, ROS rapidly induces necroptosis during Mtb infection, resulting in the release of mycobacteria into the growth-permissive extracellular milieu (Galluzzi and Kroemer, 2008; Roca and Ramakrishnan, 2013; Wang et al., 2012). Necroptosis is a type of programmed necrosis which acts as a backup to or competitor with apoptosis. A recent study found that repression of either RIP3 or MLKL could not protect cells from death but switch TNF-induced necroptosis toward a delayed RIP1-depend apoptosis (Dondelinger et al., 2013; Han et al., 2011; Remijsen et al., 2014). After TNF receptor 1 (TNFR1) is stimulated by TNF, TRADD (TNF receptor-associated death domain) binds to RIP1, TNF receptor-associated factor 2/5 (TRAF2/5) and cIAP1/2 (cellular inhibitor of apoptosis 1/2) to form the membrane-proximal super-molecular structure complex 1 (Han et al., 2011). Lys 63-linked polyubiquitination of RIP1 or TRAF2 by cIAPs results in NF-κB translocation into nucleus to induce transcription of its target genes including A20 and cylindromatosis (CYLD), both of which can deubiquitinate RIP1 (Vandenabeele et al., 2010). Deubiquitination of RIP1 or inhibition of cIAP proteins promotes the conversion of complex I to complex II, which contains RIP1, FADD, caspase-8 and TRADD (Han et al., 2011). In complex II, caspase-8 becomes activated and initiates apoptosis. If the activity of caspase-8 is abolished, RIP1 and RIP3 assemble in complex III with FADD and caspase-8 and, possibly, also TRADD. Complex III is also called necrosome, in which RIP1 phosphorylates RIP3 and further recruits MLKL, leading to necroptosis. Furthermore, activation of deubquitinating enzymes or the presence of inhibitors of ubiquitinating enzymes keeps caspase-8 inactive (Vandenabeele et al., 2010). Thus, avirulent Mtb strains are inclined to induce apoptosis, while virulent Mtb strains tend to switch apoptosis to necroptosis, which benefits pathogen and contributes to Mtb dissemination (Jayaraman et al., 2013). We deduce that during certain stages of infection, virulent Mtb strains might cause higher expression of TNF than avirulent strains to induce more necroptosis. Alternatively, virulent strains could, while avirulent strains could not, secrete a biological factor which can block caspase-8 activity to inhibit apoptosis. Meanwhile, we do not deny other interpretations for different cellular destinies caused by Mtb strains with different degree of virulence (Fig. 1).

## TYPE I IFNs FUNCTION AS A DOUBLE-EDGED SWORD DURING MTB INFECTION

IFNs can be divided into three types, including type I IFNs (IFN-α and IFN-β), type γ IFN (IFN-γ) and type λ IFNs (IFN-λ1, IFN-λ2 and IFN-λ). Type I IFNs have a pivotal position on anti-viral immune responses and are important for the host response against bacterial infection. The production of type I IFNs leads to protection against some intracellular pathogens (Du et al., 2013). Bacterial induction of type I IFNs can be mediated through the TLR-dependent recognition of bacterial products, such as lipopolysaccharide (LPS), or through the TLR-independent recognition of bacterial ligands that are delivered to the host cytosol (Gonzalez-Navajas et al., 2012; Simmons et al., 2010). After the onset of adaptive immunity, type I IFNs limit the number of myeloid cells in the lungs, in particular CD11cnegCD11b + Gr-1lo/neg macrophages, and this initial increased cell populations promote infection by providing target cells for Mtb intracellular growth (Desvignes et al., 2012). High-mobility group protein 1 (HMGB1) is a non-histone nuclear protein, which can be induced to release to outside of the cell by type I IFNs, and HMGB1 can further induce apoptosis under certain circumstances such as in the presence of LPS or polyinosinic-polycytidylic acid [poly(I:C)] stimulation (Jiang et al., 2007). In response to type I IFNs, signal transducer and stimulator of transcription1 (STAT1) can be activated and form homodimers, then those homodimers could bind to IFN-γ-activated site (GAS) enhancer elements in the promoters of IFN-stimulated genes (ISGs), resulting in the induction of pro-inflammatory cytokines or apoptotic factors such as TNF and NO (van Zoelen et al., 2009; Bordón et al., 2011; Gonzalez-Navajas et al., 2012). Besides, STAT1–STAT2 heterodimers are activated and bind to IFN regulatory factor 9 (IRF9) in the cytosol to form the IFN-stimulated gene factor 3 (ISGF3) complex, which in turn move to the nucleus to bind to IFN-stimulated response elements (ISREs) and activate antiviral or antibacterial genes such as ISGs, leading to the production of apoptosis-related factors, such as Fas, Fas ligand (FasL), caspase-4 and caspase-8 (Chawla-Sarkar et al., 2003; Gonzalez-Navajas et al., 2012). In infected macrophages, Mtb inhibits IFN-stimulated tyrosine phosphorylation of STAT1 to form homodimers and the stimulation of ISGF3 tends to decline in macrophages, while *Mycobacterium bovis* BCG can’t inhibit STAT1 homodimers (Prabhakar et al., 2003).

**Figure 2:**
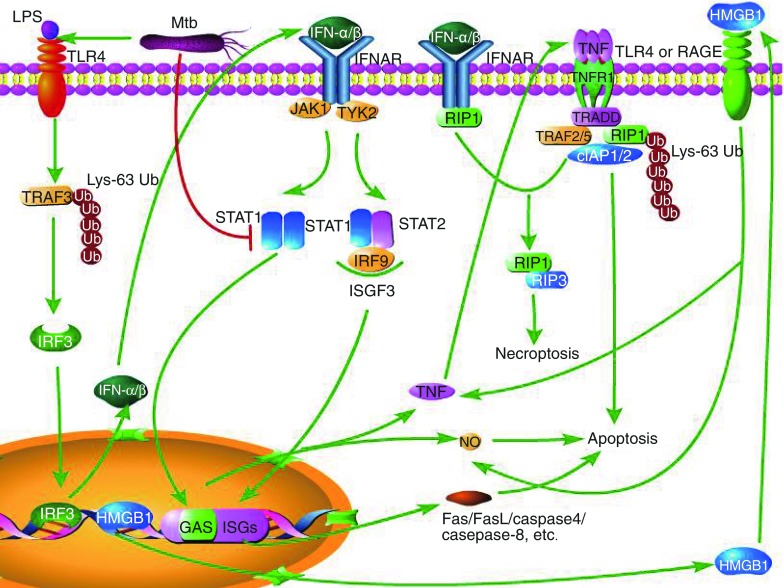
**Apoptosis and necroptosis induced by IFN-α/β**. Recognition of LPS from Mtb by TLR4 leads to Lys-63-linked polyubiquitination of TRAF3 (TNF receptor‑associated factor 3), which activates IRF3 (IFN regulatory factor 3). The activated IRF3 enters into nucleus and targets DNA, leading to the expression of IFN-α/β. IFN-α/β then binds to IFN-α/β receptor (IFNR), which activate JAK1 (Janus kinase 1) and TYK2 (non‑receptor tyrosine kinase 2). The activation of JAK1 and TYR2 can result in homodimerization of STAT1 and formation of ISGF3. STAT1 homodimers bind to GAS enhancer elements in the promoters of ISGs, which result in the production of pro-inflammatory factors such as TNF and NO, both of which are inducers of apoptosis. ISGF3 migrates to the nucleus to bind to ISGs, leading to the production of apoptosis-related factors. INF-α/β also can mediate necroptosis by inducing interaction of TNFR1 and RIP1, which promotes formation of RIP1-RIP3 and release of HMGB1 from nucleus to outside of the cell. The extracellular HMGB1 binds to TLR4 or RAGE (the receptor for advanced glycation end products) to increase the levels of TNF and NO

Type I IFNs has also been shown to exacerbate TB in mice and to be associated with disease progression in TB patients. Mtb triggers host type I interferons signaling to reduce the production of interleukin-1β (IL-1β), a critical mediator of immunity to Mtb (Novikov et al., 2011). Type I IFNs inhibit IL-1β through two different mechanisms (Guarda et al., 2011). First, type I IFN activates STAT1, and activated STAT1 directly abolishes the activity of the NOD-, LRR- and pyrin domain-containing 1 (NLRP1) and NLRP3 inflammasomes, thus caspase-1-dependent IL-1β maturation is suppressed. Second, Type I IFNs induce the production of IL-10, which binds to receptor in an autocrine manner, leading to decrease of the level of the precursor, pro-IL-1β, which requires cleavage by caspase-1 to become its active form. IL-1β can directly kill Mtb in murine and human macrophages, and it can also kill the pathogen indirectly through the recruitment of other antimicrobial effector molecules. IL-1β directly augments TNF signaling in macrophages through the up-regulation of TNF secretion and cell surface expression of TNFR1, which results in activation of caspase-3. Thus, IL-1β and downstream TNF production lead to caspase-dependent restriction of intracellular Mtb growth (Jayaraman et al.). Virulent mycobacteria induce higher level of IFN-α/β than avirulent mycobacteria species (Prabhakar et al., 2003). The increased production of type I IFNs contribute to increased susceptibility to Mtb, because IFN-α/β impairs the ability of human macrophage to control the growth of this pathogen, thus strongly enhances its replication (Bouchonnet et al., 2002). Type I IFNs could enhance the susceptibility of mice to infection caused by the intracellular pathogen *S. typhimurium* through the induction of macrophage necroptosis (Liang and Qin, 2013), thus type I IFNs may also cause macrophage necroptosis during Mtb infection, which benefit pathogen and contribute to Mtb dissemination (Jayaraman et al., 2013). The signaling pathways leading to IFN-α/β-induced apoptosis and necroptosis are shown in Fig. 2.

## VITAMIN D HELPS COMBAT TB VIA INDUCTION OF CATHELICIDIN AND AUTOPHAGY

**Figure 3:**
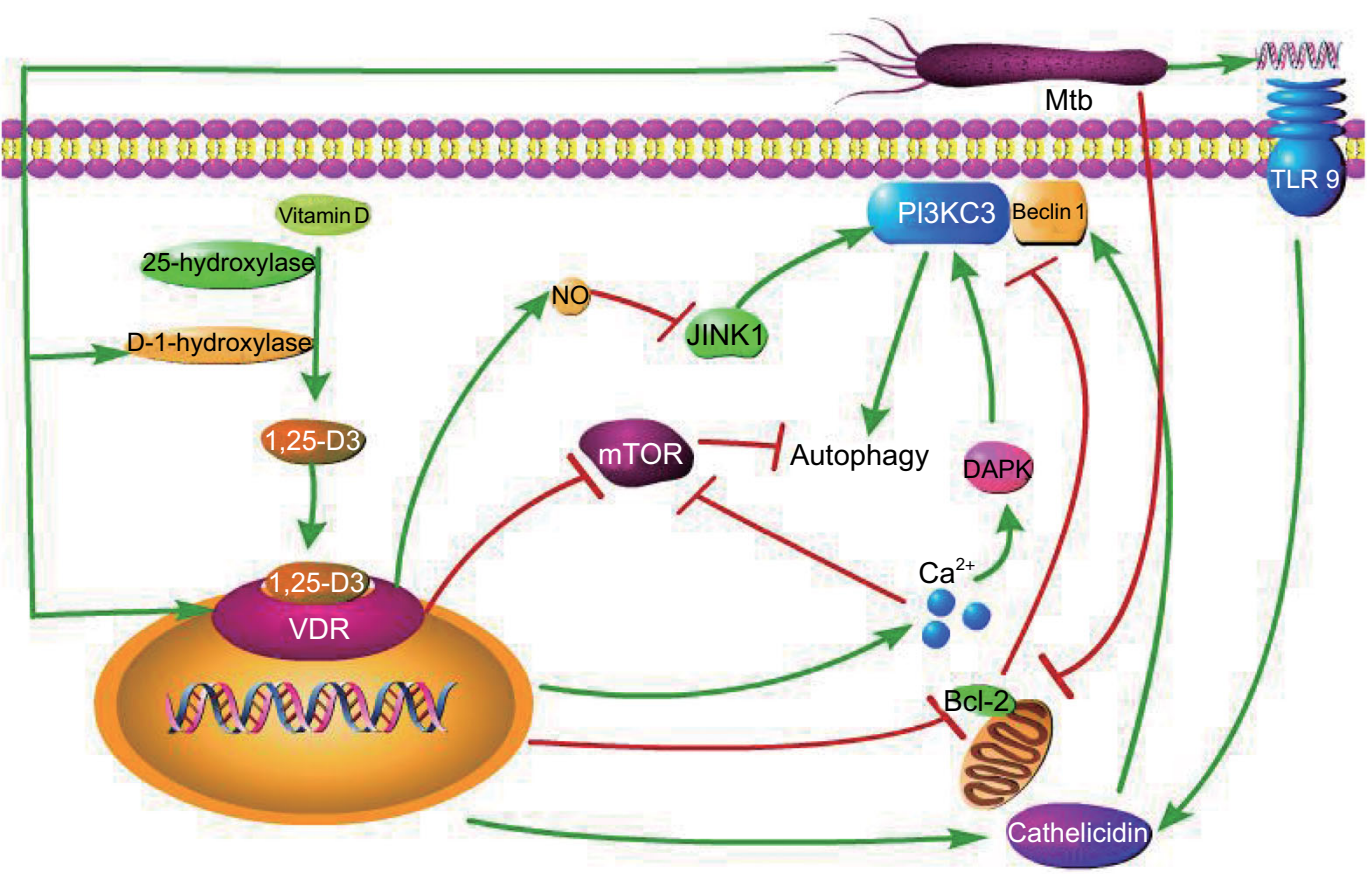
**Autophagy induced by Vitamin D**. After being taken up by the cell, Vitamin D is catalyzed into 1,25-D3 by 25-hydroxylase and D-1-hydroxylase. 1,25-D3 binds to VDR to activate autophagy through down-regulating mTOR and Bcl-2, and increasing NO, Ca^2+^ and cathelicidin. Both JNK1 and DAPK (death-associated kinase) can mediate autophagy by regulating the activity of PI3KC3. NO impairs the activity of JNK1, and Ca^2+^ enhances the activity of DAPK. Bcl-2 can bind to Beclin 1 to inhibit the activity of PI3KC3. Cathelicidin mediates autophagy through increasing Beclin 1. Mtb can induce autophagy by the up-regulation of D-1-hydroxylase, VDR and cathelicidin, and the down-regulation of Bcl-2. Cathlicidin can be induced by Mtb DNA-mediated stimulation of TLR9

For a long time, Vitamin D is considered critical for bone metabolism (Mata-Granados et al., 2013). In recent years, accumulating studies have demonstrated that Vitamin D also plays a vital role in the regulation of innate immunity in human macrophages and control of proliferation of Mtb. TLR activation up-regulates the expression of Vitamin D receptor (VDR) and Vitamin D-1-hydroxylase, leading to the induction of a peptide, namely cathelicidin, which can kill intracellular Mtb (Liu et al., 2006). During early infection, Mtb DNA can strongly stimulate TLR-9 to induce cathelicidin LL-37 (Rivas-Santiago et al., 2008). Cathelicidin not only has direct antimicrobial activity, but also serves as mediator of 1,25-dihydroxyvitamin D3 (1,25-D3)-induced autophagy by up-regulation of Beclin (Wu and Sun, 2011). Upon Vitamin D treatment, the expression of mitogen-activated protein kinase phosphatase-1 (MKP-1) is significantly up-regulated, by which Vitamin D inhibits LPS-induced p38 activation and JNK, leading to subsequent inhibition of pro-inflammatory cytokine production in monocytes and macrophages (Zhang et al., 2012). Increased calcium absorption mediated by the effect of Vitamin D3 on the VDR can activate autophagy through various calcium-dependent kinases and phosphatases, while Vitamin D3 can itself down-regulate the expression of Bcl-2 and mammalian target of rapamycin (mTOR), a negative regulator of autophagy and Bcl-2. mTOR is a negative regulator of autophagy, which binds to Beclin 1 and abolishes the activity of mammalian class III PI3PK complex (PI3KC3) (Mouli and Ananthakrishnan, 2014; Wu and Sun, 2011). Mycobacterial lipoprotein LpqH stimulates TLR2/1/CD14, triggering TLR2/1/CD14-Ca^2+^-AMPK-p38 MAPK pathways, contributing to CCAAT/enhancer-binding protein(C/EBP)-β-dependent expression of D-1-hydroxylase and cathelicidinis, which play an essential role in LpqH-induced autophagy (Shin et al., 2010). 1,25-D3, the active form of Vitamin D, generally boosts infection-stimulated cytokine and chemokine responses and induces the co-localization of mycobacterial phagosomes with autophagosomes in human macrophages and synthesis of nitric oxide (NO) synthase that subsequently produce NO (Rockett et al., 1998; Veyrier et al., 2009; Yuk et al., 2009). NO can induce mitochondrial permeability transition and promote apoptosis (Brune, 2003), and it can also impair autophagy by inhibiting the activity of S-nitrosylation substrates, JNK1 and IKKβ (Fabri et al., 2011). 1,25-D3 also can enhance IL-1β expression via a direct transcriptional mechanism (Verway et al., 2013). Furthermore, Vitamin D is required for IFN-γ-mediated antimicrobial activity of human macrophages (Sarkar et al., 2011). The signaling pathways involved in Vitamin D-induced autophagy are shown in Fig. 3. A study has shown that TB patients under normal treatment supplemented with 0.25 mg Vitamin D per day reduced the time for sputum smear conversion from acid fast bacteria (AFB) positive to AFB negative (Nursyam et al., 2006). Ultraviolet-B ray from sunlight triggers synthesis of Vitamin D, thus TB tends to occur during the colder season when cutaneous synthesis of Vitamin D from sun exposure is reduced and serum Vitamin D levels are lower (Fuchs and Steller, 2011). Humans who lack Vitamin D3 are more likely to be infected with Mtb. Because of skin melanin content and diminished ultraviolet (UV) light-dependent cutaneous Vitamin D3 synthetic capacity, African-Americans have significantly decreased level of serum 1,25-D3, and have increased susceptibility to TB (Liu et al., 2006). Therefore, supplementing with certain amount of Vitamin D and exposure to sunlight helps combat TB.

## SUMMARY AND PERSPECTIVES

**Figure 4:**
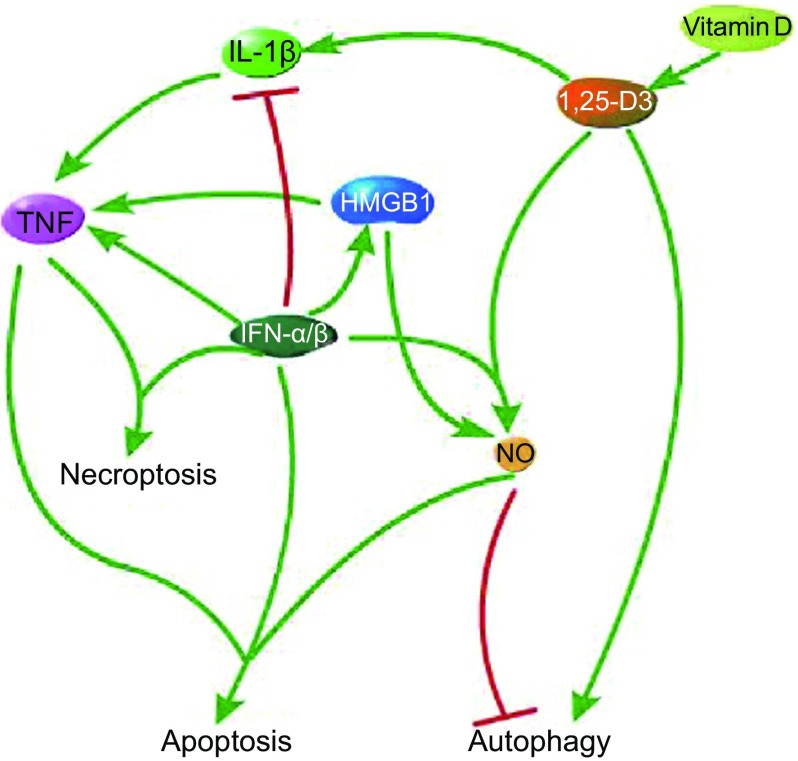
**The signaling pathways that involve TNF, Type I IFNs and Vitamin D are inter-connected**. TNF can induce both apoptosis and necropotosis. 1,25-D3, the active form of Vitamin D, can up-regulate IL-1β and NO, and induce autophagy. NO can induce apoptosis and impair autophagy as well. Type I IFNs (IFN-α/β) can induce necroptosis and translocation of HMGB1 from nucleus to cytoplasm to induce apoptosis. In addition, TNF α/β can down-regulate IL-1β

Different cellular fates of Mtb-infected macrophages are regulated by a number of immune-regulatory molecules and multiple inter-connected cellular pathways. Programmed cell death plays a fundamental role that helps maintain cellular homeostasis, shapes the growth of organism, and provides protective immunity against invading pathogens (Fuchs and Steller, 2011). Mtb can induce infected macrophages to secrete pro-inflammatory cytokine TNF, which can trigger caspase-8-dependent apoptosis. If the activity of caspase-8 is inhibited, apoptosis would be switched to necroptosis. Virulent Mtb strains induce higher level of TNF than avirulent or attenuated strains, and excessive TNF induce necroptosis, thus virulent strains tend to cause necroptosis. In the face of apoptosis, virulent Mtb strains have developed a capacity to disrupt this mechanism, such as expression of anti-apoptotic Bcl-2 family. Type I IFNs function as a double-edged sword that both control pathogens and exacerbate pathogenicity. On the one hand, type I IFNs restrict the number of myeloid cells in the lungs during early adaptive immunity, cause translocation of HMGB1 from nucleus to outside of the cell to induce apoptosis, and result in the production of pro-inflammatory cytokines or apoptotic factors. On the other hand, type I IFN lowers the level of IL-1β, increases susceptibility to Mtb, and even may lead to necroptosis. IL-1β can directly kill Mtb in murine and human macrophages, up-regulate TNF and further induce apoptosis. 1,25-D3, the active form of Vitamin D, can induce cathelicidin, which can directly kill intracellular Mtb and activate autophagy. Besides, 1,25-D3 can also induce the synthesis of NO synthase to produce NO and up-regulate IL-1β. Thus the signaling pathways that involve TNF, Type I IFNs and Vitamin D are inter-connected (Fig. 4). Just like the way that many food chains constitute a food web, multiple signaling pathways form a signaling network in macrophages. The more complex the signaling network, the greater the stability of the cellular function. As a result of co-evolution, the battles between the pathogen and the host have never stopped and will probably last forever. During those long-lasting battles, host cells try to keep their stable function through the complex signaling network, while the pathogen struggles to survive in the host through disrupting those cellular pathways.

Pathogens also possess a signaling network, though not as complex as that of the host. When one pathway is inhibited, other alternative pathways can compensate for the normal function of the pathogen, which is one of the major reasons for pathogen resistance toward antibiotics. Thus, in recent years, network-based computational biology, with the emphasis on bio-molecular interactions and omics-data integration, has been adopted in the development of drug cocktails for the treatment of intractable pathogens such as Mtb. Such kind of treatment strategy could not only alleviate the problems of the recurrent emergence of drug resistance but also reveal their synergistic effects (Andersen, 1997; Wu et al., 2013). We hold an opinion that multiple drugs should be aimed at relevant sites instead of arbitrary targets so that the pathogens could not escape from alternative pathways. In addition, the multi-drug therapy should enhance beneficial signaling pathways while abolishing harmful pathways in host cells in order to alleviate side effects. For example, most vaccine candidates for TB are screened for their capacity to induce multi-functional T cells capable of producing TNF, since high TNF production could cause necroptosis (van Heijst and Pamer, 2013), thus repression of RIP3 or MLKL should be considered to switch TNF-induced necroptosis toward RIP1-dependent apoptosis in the presence of excessive TNF. With increasing knowledge on molecular details involved in Mtb-host interactions as well as mechanisms of drug action, we firmly believe that more effective multi-drug therapies for the treatment of Mtb including drug-resistant Mtb would be more widely adopted in the near future.

## ABBREVIATIONS

AFB: acid fast bacteria
Bcl-2: B-cell lymphoma/leukemia 2
C/EBP: CCAAT/enhancer-binding protein
cIAP1/2: cellular inhibitor of apoptosis 1/2
DAPK: death-associated kinase
FasL: Fas ligand
Drp1: dynamin-related protein-1
GAS: IFN-activated site
IRF9: IFN regulatory factor 9
ISGF3: IFN-stimulated gene factor 3
ISREs: IFN-stimulated response elements
MDR: multidrug-resistant
ISG: IFN-stimulated gene
IFNR: IFN-α/β receptor
IRF3: IFN regulatory factor 3
JAK1: Janus kinase 1
MKP-1: mitogen-activated protein kinase phosphatase-1
MLKL: Mixed lineage kinase domain-like protein
MyD88: myeloid differentiation factor 88
Mtb: *Mycobacterium tuberculosis*
mTOR: mammalian target of rapamycin
NLRP1: NOD-, LRR- and pyrin domain-containing 1
NO: nitric oxide
PAMPs: pathogen-associated-molecular patterns
PGAM5: phosphoglycerate mutase family member 5
PI3KC3: mammalian class III PI3PK complex
poly(I:C): polyinosinic-polycytidylic acid
RAGE: receptor for advanced glycation endproducts
RIP1: receptor interacting protein 1
RIP3: receptor interacting protein 3
ROS: reactive oxygen species
STAT1: signal transducer and stimulator of transcription1
TB: tuberculosis
TLRs: Toll-like receptors
TNF: tumor necrosis factor
TNFR1: TNF receptor 1
TRADD: TNF receptor-associated death domain
TRAF2/5: TNF receptor–associated factor 2/5
TRAF3: TNF receptor‑associated factor 3
TYK2: non‑receptor tyrosine kinase 2
VDR: Vitamin D receptor
XDR: extensively-drug resistant
